# Repurposing Anti‐diabetic GLP‐1 Receptor Agonists for the Treatment of Alzheimer's Disease

**DOI:** 10.1002/alz70855_101740

**Published:** 2025-12-28

**Authors:** Amaan Ali Mohammed

**Affiliations:** ^1^ Cambridge Centre for International Research (CCIR), Dublin, CA, USA

## Abstract

**Background:**

Alzheimer's Disease (AD) is a type of neurodegenerative disease marked by cognitive decline, memory impairment, and various neuropsychiatric symptoms that significantly impact patients and the healthcare system. Current AD treatments focus on symptom management rather than addressing the underlying disease mechanisms. The pathogenesis of AD involves genetic, environmental, and lifestyle factors, with amyloid‐beta plaques and neurofibrillary tangles being primary pathological features. Glucagon‐like peptide‐1 receptor agonists (GLP‐1 RAs), primarily used in type 2 diabetes mellitus (T2DM) and obesity management, have shown potential neuroprotective effects, including reducing neuronal apoptosis and neuroinflammation and improving cognitive function in AD models.

**Method:**

This comprehensive review covers four GLP‐1 RAs (semaglutide, liraglutide, exenatide, and lixisenatide). It analyzes the outcomes of their in vivo studies to illustrate the potential of GLP‐1 RAs in serving as effective first‐line treatment for AD patients.

**Result:**

Findings from the semaglutide study suggest that GLP‐1 RA enhances glucose metabolism dysfunction in AD models by regulating the SIRT1 signaling pathway, which leads to improved memory and decreased amyloid plaques. The Liraglutide study also observed the reversal of key AD pathologies, decreased inflammation, and reduced amyloid plaque deposition in early‐stage AD models. Exenatide can cross the blood‐brain barrier and activate GLP‐1 receptors in the CNS, resulting in neuroprotection. Also, findings from the Lixisenatide study show an activation of the PKA‐CREB signaling pathway to prevent amyloid plaque formation.

**Conclusion:**

The GLP‐1 RAs exhibit neuroprotective mechanisms through anti‐inflammatory actions, enhancing synaptic function and reducing amyloid plaque formation. These findings warrant further clinical trials to establish their efficacy and safety in humans. The comparison of in vivo studies highlights the positive impact of GLP‐1 RAs on cognition, learning, memory, and reducing AD pathology, suggesting their potential as first‐line treatments for AD patients.

